# Cerebrospinal fluid-stem cell interactions may pave the path for cell-based therapy in neurological diseases

**DOI:** 10.1186/s13287-018-0807-3

**Published:** 2018-03-09

**Authors:** Chao Ren, Peiyuan Yin, Neng Ren, Zhe Wang, Jiahui Wang, Caiyi Zhang, Wei Ge, Deqin Geng, Xiaotong Wang

**Affiliations:** 1 Department of Neurology, Affiliated Yantai Yuhuangding Hospital of Qingdao University, Yantai, 264000 China; 2 Department of Blood Supply, Yantai Center Blood Station, Yantai, 264000 China; 3 Department of Inervention Therapy, Affiliated Hospital of Xuzhou Medical University, Xuzhou, 221004 China; 4 Department of Clinical Laboratory, Affiliated Yantai Yuhuangding Hospital of Qingdao University, Yantai, 264000 China; 5 Department of Central Laboratory, Affiliated Yantai Yuhuangding Hospital of Qingdao University, Yantai, 264000 China; 6 Department of Neurology, Affiliated Hospital of Xuzhou Medical University, Xuzhou, 221004 China

**Keywords:** Cerebrospinal fluid, Stem cells, Induced differentiation, Neural stem cells, Stem cell transplantation therapy

## Abstract

Recent studies have suggested that the regulation of endogenous neural stem cells (NSCs) or transplanting of exogenous nerve cells are the newest and most promising methods for the treatment of dementia and other neurological diseases. The special location and limited number of endogenous NSCs, however, restrict their clinical application. The success in directional differentiation of exogenous stem cells from other tissue sources into neural cells has provided a novel source for NSCs. Study on the relative mechanisms is still at the preliminary stage. Currently the induction methods include: 1) cell growth factor induction; 2) chemical induction; 3) combined growth factor-chemical induction; or 4) other induction methods such as traumatic brain tissue homogenate, gene transfection, traditional Chinese medicine, and coculture induction. Cerebrospinal fluid (CSF), as a natural medium under physiological conditions, contains a variety of progrowth peptide factors that can promote the proliferation and differentiation of mesenchymal stromal cells (MSCs) into neural cells through the corresponding receptors on the cell surface. This suggests that CSF can not only nourish the nerve cells, but also become an effective and suitable inducer to increase the yield of NSCs. However, some other studies believed that CSF contained certain inhibitory components against the differentiation of primary stem cells into mature neural cells. Based on the above background, here we review the relative literature on the influence of the CSF on stem cells in order to provide a more comprehensive reference for the wide clinical application of NSCs in the future.

## Background

The clinical treatment of neurological diseases, especially degenerative diseases of the nervous system, has always proved very difficult. Only by symptomatic treatment can we delay the progression of the disease as much as possible; however, the treatment efficacy is generally dissatisfactory, not to mention the hope of a complete cure. The reason for this is that once the nerve cells are damaged and degenerated they cannot self-repair. Can neurological function loss caused by neurological diseases be improved or even cured by nerve regeneration or functional replacement by neighboring nerves? The answer is positive. Research over recent years has found that the nerves of patients with neurological diseases have certain self-repair potential after nervous system injury [1], and proliferative neural stem cells (NSCs) can still be found in adult nervous tissues. Therefore, the latest and most promising method to treat neurological diseases is by artificial intervention and regulation of endogenous NSCs, which can promote their proliferation and differentiation, or by nerve cell transplantation to promote the repair of central nervous system injury. Because of the special location and limited number of endogenous NSCs, their clinical application has been restricted. Although embryonic (including umbilical cord) stem cells are good source libraries of exogenous NSCs and these have made great progress in animal models, their clinical applications have been strictly restricted due to reproductive ethical problems [2]. Former president of the United States, Mr. Bush, issued an injunction in 2001 to prohibit the federal government from funding human embryonic stem cell research. The recent trend of induced pluripotent stem cell (iPSC) research no longer has this reproductive ethics problem. However, since it introduces some transcription factors into animal or human somatic cells through gene transfection which leads to the direct reconstruction of somatic cells into embryonic stem cell-like pluripotent cells, the risk of carcinogenesis is higher than in normal cells. In addition, it is technically demanding and complicated in operation, which has restricted its clinical application. The discovery of the pluripotency of bone marrow-derived mesenchymal stromal cells (BM-MSCs) and the success of directional differentiation of nerve cells has provided a new source of NSCs [3]. The study on the mechanism of BM-MSCs differentiating into neural cells is still at a preliminary stage. At present, the main induction methods include: 1) cell growth factor induction by epidermal growth factor (EGF), basic fibroblast growth factor (bFGF), nerve growth factor (NGF), and so forth [4]; 2) chemical induction by β-mercaptoethanol (B-ME), dimethyl sulfoxide (DMSO), butylated hydroxyanisole (BHA), and so forth [5]; 3) growth factor and chemical combined induction (Woodbury et al. [6] used B-ME, DMSO, and bFGF combined induction while Kögler et al. [7] utilized NGF, bFGF, DB-cAMP, isobutylmethyl xanthine, and RA for combined induction of BM-MSCs into nerve cells in vitro); and 4) other methods such as traumatic brain tissue homogenate [8], gene transfection [9], traditional Chinese medicine (Baicalin, Salvia miltiorrhiza, and so forth) [10], coculture, and conditioned growth medium close to the physiological state. Cytokines are widely used inducers due to their extensive function in neural nutrition, antifree radicals, reducing calcium overload, and inhibiting the expression of nitric oxide synthase. Among them, EGF and bFGF are most representative as they are not only strong polypeptide factors for promoting cell growth, but also important mitogens which promote the proliferation and differentiation of BM-MSCs through corresponding receptors on the cell surface [11]. However, these are all exogenous substances that more or less impose certain risks. It has been one of the goals of stem cell researchers to find inducers that are close to the microenvironment of the human body, preferably the human body’s own secretion. Cerebrospinal fluid (CSF), as a natural medium at the physiological state, is the best candidate [12]. CSF is mainly secreted by the lateral ventricle choroid plexus epithelial cells. It is a colorless and transparent liquid that contains a variety of electrolytes, proteins, sugars, and various growth factors such as the brain-derived and gliocyte-derived neurotrophic factors [13]. These factors in CSF can promote the proliferation and differentiation of MSCs into nerve cells through the corresponding receptors on the cell surface, suggesting that the differentiation of MSCs is tissue-specific and that the tissue microenvironment can induce its directional differentiation [14]. This indicates that CSF can not only nourish the nerve cells, but can also act as an effective and suitable inducer thus providing a new source of NSCs [15]. On the other hand, some studies have suggested that the inhibitory components in CSF might suppress the differentiation of related primary stem cells into mature nerve cells [16]. Based on the above information, here we review the reported effects of CSF on stem cells in the worldwide literature in order to provide references for the future clinical application of NSCs.

### Effects of CSF on MSCs

Yan and colleagues reported that BM-MSCs displayed neuronal morphology 4–5 days after autologous CSF induction, differentiated into neurons, astrocytes, and oligodendrocytes, and exhibited corresponding characteristic structure and biological features [17]. Our group had similar observations in our previous studies, and the same effect was also observed in umbilical cord blood mesenchymal stromal cells (UCB-MSCs) [3, 12, 18–25]. Farivar et al. [26] also confirmed that CSF could induce the differentiation of UCB-MSCs into neuronal cells, although the concentration of CSF used for the induction and the time needed for differentiation was slightly different. These studies suggested that MSCs can grow in CSF and maintain their potential of differentiation into neural cells. Shen et al. [27] also showed that the growth characteristics of MSC in CSF and in general medium were similar. As we have shown, the number of various hemocytes in the suspension decreases during the rapid growth of MSCs suggesting suppressed proliferation or differentiation of hematopoietic stem cells and suggesting that CSF favors the growth of MSCs over hematopoietic stem cells which contributes to the isolation and culture of MSCs from bone marrow or umbilical cord blood. However, attention should be paid to the protocols for CSF culture and induction because of the potential of MSCs to differentiate into different tissue cells in different culture media. Therefore, different culture media formulae are needed based on the purpose of the culture.

The mechanism of CSF-induced differentiation of MSCs into nerve cells is rarely discussed, but it is theoretically proposed that the microenvironment within the brain or the spinal cord provides the necessary conditions for the induced directional differentiation of MSCs, although the specific functioning elements are still unknown. Recently Zhu et al. [28] identified that CSF regulated the proliferation and migration of stem cells through insulin-like growth factor 1 (IGF-1), while Glage et al. [29] suggested that glucagon-like peptide 1 (GLP-1) might be an important regulator for this process. Some researchers [30–33] believe that it is the result of the direct interaction between the choroid plexus cells and MSCs in CSF, or the fusion of these two types of cells. Other scholars [34, 35] proposed that the microecological signals of choroid plexus cells might be an important factor regulating the differentiation and migration of stem cells, and that PRDM16 might be an important regulator in this signaling pathway [35]. In addition, a study [36] found that the CSF of amyotrophic lateral sclerosis patients could also promote MSCs to differentiate into neuron-like cells. Currently, the study of the influence of the CSF on MSCs is not limited to induction experiments—there have been reports on the use of nerve cells obtained from induced MSCs in clinical treatment and which have shown certain effects [3, 18, 20, 25, 37].

### Effects of CSF on embryonic stem cells (ESCs)

Bian et al. [38] induced the differentiation of human embryonic stem cells (hESCs) into neuron-like cells (such as neurons, astrocytes, and oligodendrocytes) using healthy human CSF, but the proportion of each cell type was different. The proportion of glial cells was higher, likely because CSF contains more factors that preferably induce the differentiation of stem cells into glial cells. Chen et al. [39] used bloody CSF to induce ESCs and obtained a higher proportion of glial cells which might be related to the increase of stimulating factors for glial cell proliferation in CSF after traumatic brain injury. During the study of migration and differentiation of human fetal brain NSCs in developmental CSF, Yin et al. [40] found that in the embryo there were large differences in the information substances secreted by ESCs at different developmental stages, which in turn affected the components of the CSF since the blood-brain barrier was not yet formed and the brain was therefore in an open state. Furthermore, the different active constituents in the CSF could also affect the development and differentiation of ESCs. This further confirmed that the production of glial cells may be closely related to the specifics of the CSF environment.

Unlike the above research on obtaining glial cells from induced ESCs, Xu et al. [41] used certain concentrations of ascorbic acid to induce the differentiation of ESCs, and then adult CSF was used instead to induce the directional differentiation of embryonic brain stem cells into dopaminergic neurons. Zappaterra et al. [42] identified the CSF fluid pressure to be an important contributing factor to the differentiation and migration of ESCs, while Martin et al. [43] proposed FGF2 to be an important factor in CSF-induced ESC differentiation. Through genetic analysis, factors in CSF have been confirmed to greatly influence the early embryonic development process, especially the differentiation and formation of nerve cells [44]. Kiiski et al. [45] found that the CSF from healthy people promoted the differentiation of hESCs into neurocytes and the formation of a neural network with spontaneous activity. However, another study suggested that adult CSF did not support neurogenesis of ESCs [46]. There have been other reports that CSF suppressed the differentiation of ESCs into neurons but promoted their differentiation into glial cells [47]. The inconsistency of these results might be due to the different sources of ESCs and CSF used in the studies.

### Effect of CSF on NSCs

Li [48] found that after spinal cord injury the changes in the CSF components affected the proliferation and differentiation of endogenous NSCs in the spinal cord. Another comparison study [49] found that: 1) NSCs can survive, proliferate, and differentiate in the bloody CSF and hydrocephalus clear CSF; 2) the adherent differentiation of NSCs in the traumatic bloody CSF was faster than that in the hydrocephalus clear CSF, and the proportion of adherent differentiation was also higher; and 3) there was difference in the cell types of NSC differentiation in traumatic bloody CSF and hydrocephalus clear CSF. NSCs were prone to differentiate into glial cells in traumatic bloody CSF and into neurons in hydrocephalus clear CSF. Teng et al. [50] discovered that the CSF of ischemic rats could promote the survival of NSCs in vitro and induced the differentiation of NSCs into neurons and astrocytes. Nozaki et al. [51] studied the CSF of patients with subarachnoid hemorrhage and identified bloody CSF to be an effective stimulant to activate and promote the proliferation and differentiation of endogenous NSCs. Haines et al. [52] reported that the CSF of multiple sclerosis patients induced transcriptional changes in oligodendrocyte progenitor cells of the NSCs. The above research suggested that CSF under morbid conditions might be an important factor to initiate patient’s self-endogenous nerve repair [53, 54] but that during the repair there was a difference in the differentiation direction of NSCs [55]. In most cases NSCs mainly differentiate into glial cells [15] and less frequently predominantly into neuron-like cells [56]. Thus a current research hotspot in treating neurological diseases with induced endogenous stem cells is how to obtain the desired cells for clinical treatment, currently drug-containing CSF, and especially traditional Chinese medicine CSF pharmacology [57]. Our research group is currently exploring the treatment of dementia using the transplantation of EGb761 CSF pharmacology-mediated circulating stem cells.

## Conclusions: Problems and prospects

Currently, a growing number of studies (Table 1) have suggested that the CSF-stem cell interaction is a potential for the treatment of neurological diseases [16]. This is because CSF not only provides the microenvironment for stem cell growth [13] and acts as the regulator for stem cell differentiation [58], but it also conducts cell signals to stimulate self-healing [59]. However, in the absence of unified and/or preferential stem cell culture and identification methods, and specifications and/or guidelines for stem cell transplantation approaches, conditions and time windows, there will inevitably be some negative reports and exaggerated propaganda, such as the controversial “stem cell tourism” [60]. What should we do in the face of this embarrassment? Sometimes it might be “better to leap before looking” [61]. As Nobel Prize winner Martin Evans said during his interview with *Life Times*, the key point of stem cell research is to apply experimental results to clinical practice. Based on previous experiences [3, 18], we propose that the synchronous treatment and acquisition of CSF through CSF circulating transplantation broke the boundary between stem cell induction and treatment, and circumvented the limitation of transplantation routes including stereotactic injection, operation injection, and intravenous infusion. It is an effective and clinically feasible individualized stem cell transplantation treatment mode. However, this is based on only small-scale clinical research. Further multicenter and large-scale randomized control trials are needed to solve the following major questions: 1) What is the specific substance(s) in CSF that induces the differentiation of stem cells? 2) What is the proportion of target cells derived from CSF-induced differentiation, and how to increase this proportion? 3) Why are there were differences in obtaining functional stem cells from different sources of mesenchymal stromal cells [62, 63]? 4) How do we prevent stem cells senescence and maintain multiple attributes [64–66]? 5) How many stem cells should be used for transplantation? 6) How do we control the proliferation, differentiation, migration, and tumorigenesis of the stem cells after implantation into the nervous system? 7) Why are there are no standard guidelines for the indications, routes, and timing of transplantation, or the evaluation criteria of treatment results? In this regard, we agree with the opinion of responsible professionals and experts that basic research and applied basic research of CSF-induced stem cells should be encouraged, while standardized clinical research of a scientific nature can be performed in those hospitals with appropriate facilities. Without confirmed results from relevant research, CSF-induced stem cells are currently not recommended for large-scale clinical application, and for-profit marketing and exaggerated commercialized propaganda should be prohibited.

**Table 1:** Summary of the important studies on the effects of cerebrospinal fluid on stem cells

Study	Source of CSF	Primitive cell type	Cell type after induction	Changes after induced by CSF	Cell identification markers	Transplantation (Yes or No)
Pandamooz et al., 2013 [15]	Allogeneic-rats	Endogenous neural stem cell (allogeneic-rat)	Neuron-like cells	CSF promoted the differentiation of NSCs into neuron-like cells, with the majority to be glial cells and less neurons	Nestin, β-tubulin, GAFP, genes	No
Yan et al., 2013 [17]	Autologous-human (total hip arthroplasty patients)	BM-derived MSCs (autologous-human: total hip arthroplasty patients)	NSCs	Nestin expression from 6 h, peaked at 24 h; NSE expression from 12 h, peaked at 48 h	Nestin, NSE, NF, GFAP	No
Ren et al., 2015 [18]	Autologous-human (neurodegenerative disease patients)	BM-derived (autologous-human: neurodegenerative disease patients) and umbilical (allogeneic-human: full-term healthy neonate by eutocia) MSCs	NSCs	N/A	N/A	Yes, clinical trial
Ye et al., 2009 [19]	Autologous-human (healthy voluntary donors)	BM-derived (autologous-human: healthy voluntary donor) and umbilical (allogeneic-human: full-term healthy neonate by eutocia) MSCs	NSCs	BM-MSCs semiadhered by 24 h, completely or loosely adhered by 48 h with budding, formed spindle-shaped cells with pseudo-foot by 72 h, formed large colonies by 1 week, whorled arrangement by 10 days; umbilical MSCs semiadhered by 24 h, loosely adhered with little budding by 48 h, spindle-like change by 72 h, formed small colonies by 14 days, whorled arrangement by 21 days	CD34, CD44, CD45, CD90, β-tubulin, NSE, NF, GFAP	No
Ye et al., 2015 [20]	Allogeneic-human (healthy voluntary donors)	BM-MSCs (allogeneic-rat)	Neuron-like cells	N/A	N/A	Yes, animal experiments (rats)
Feng et al., 2015 [21]	Allogeneic-human (healthy voluntary donors)	BM-MSCs (allogeneic-rat)	Neuron-like cells	N/A	N/A	Yes, animal experiments (rats)
Ye et al., 2011 [22]	Allogeneic-human (healthy voluntary donors)	BM-MSCs (allogeneic-human: healthy voluntary donor)	Neuron-like cells	After 24 h of induction cells exhibited a significant morphological change including soma retraction and transparency. After 3 days, a number of neurites were formed. The soma of 4-day cultures gradually formed a tapered, triangular and irregular shape. The soma of 7-day cultures was similar to the dendrite and axon-like structure of astrocytes	β-Tubulin, GFAP	No
Ren et al., 2013 [23]	Allogeneic-human (healthy voluntary donors)	BM-derived (allogeneic-human: patients with written informed consent for special treatment) and umbilical (allogeneic-human: full-term healthy neonate by eutocia) MSCs	NSCs	Low β-tubulin positive rates in IHC and IF by 6 h, highest β-tubulin fluorescence and positive rates at 72 h; high GAFP positive rates in IHC and IF that peaked at 48 h, and eventually dropped afterwards, positive double fluorescence staining by 72 h	CD34, CD44, CD45, CD90, β-tubulin, GAFP,	No
Chen et al., 2016 [24]	Allogeneic-human (healthy voluntary adult donors)	BM-derived (allogeneic-rat)	Neuron-like cells	Adherence from 12 h, most cells adhered after 24 h with long spindle shape, apparent colonies formed by 72 h, typical fibroblast-like cells appeared after 6 days	CD29, CD45, CD54, CD90, GAFP, NSE	No
Ye et al., 2016 [25]	Allogeneic-human (healthy voluntary adult donors)	BM-derived (allogeneic-rat)	Neuron-like cells	At day 4 postinduction by CSF, cells were digested with 0.25% trypsin and resuspended to a concentration of 1 × 10^7^ cells/ml	NSE, GFAP, BRDU,	Yes, animal experiments
Farivar et al., 2015 [26]	Allogeneic-human (normal children)	Umbilical (allogeneic-fresh human umbilical cords)	Neuron-like cells	No significant cell death after CSF treatment; a remarkable increase in Nestin expression over 21 days; MAP2 showed a delayed expression on day 21; GFAP is expressed before MAP2, GFAP expression is higher than MAP2 expression on day 14	Nestin, MAP2, GFAP	No
Shen et al., 2011 [27]	Allogeneic-human (healthy voluntary donors)	BM-derived (allogeneic-rat)	Neuron-like cells (small round cell)	After adherence by 24 h, the number of adherent cells continually increased, with long spindle shapes, exhibited typical fibroblast morphology after ~ 6 days	CD29, CD45, CD71, CD90, CD106, NSE	No
Zhu et al., 2015 [28]	Allogeneic-human	Adipose MSCs (allogeneic-human), fetal neural progenitor cells (allogeneic-human)	Stem cells	Human CSF promoted proliferation, inhibited apoptosis, increased the migration speed and distance of hAMSCs and hfNPCs, enhanced their migration capacity to GBM conditioned media; IGF-1 in human CSF affected the apoptosis and proliferation of hAMSCs and hfNPCs, enhanced the migration capacity and affect the expression of CXCR4 in hAMSCs and hfNPCs	CD31, CD34, CD45, CD73, CD90 and CD105 via flow cytometry. NPCs were stained with Nestin, GFAP and Tuj-1.GBM	No
Glage et al., 2011 [29]	Allogeneic-human	BM-derived MSCs (allogeneic-human)	Stem cells	GLP-1 CSF concentrations can improve stem cell viability	N/A	Yes, animal experiments (cats)
Yang et al., 2009 [30]	Autologous-rat	BM-derived (allogeneic-rat)	NSCs	After transplantation of BM-MSCs, CSF stimulated the differentiation into NSCs, the effect of which was more significant when transplanted earlier, cells generally adhered to the bottom of the plates by 72 h, with cell synapses	NF, Nestin	Yes, animal experiments (rats)
Wang et al., 2012 [36]	Allogeneic-human (amyotrophic lateral sclerosis patients)	BM-derived (allogeneic-rat)	Neuron-like cells	No change on day 1, cells started to shrink from day 4 and cell synapses appeared, synapses increased on day 7	CD29, CD34, CD44, CDE45, NSE, Nestin	No
Kim et al., 2015 [37]	Allogeneic-human	UCB (allogeneic-human)	NSCs	After CSF induction, UCB-MSCs enhanced the synaptic regeneration of NSCs in the hippocampus and promoted the clearance of Aβ42	N/A	Yes, animal experiments (rats)
Bian et al., 2003 [38]	Allogeneic-human: (healthy voluntary donors)	Embryo (allogeneic-human)	Neuron-like cells	Adhered after 24–48 h, proliferated and differentiated after 2 days, stabilized after 3 weeks	NF, GFAP, Galc	No
Chen et al., 2011 [39]	Allogeneic-human (healthy voluntary donors), allogeneic-human (closed brain contusion patients)	Embryo (allogeneic-human)	Neuron-like cells	Adhered and differentiated after 2–3 h, adherence peaked by 10 h with increased differentiation	GFAP, MAPR, Nestin	No
Yin et al., 2010 [40]	Allogeneic-human (cerebral palsy children)	Embryo (allogeneic-human)	Neuron-like cells	Cell migrated from 6 hrs, cell clusters formed after 4 days	GFAP, NF, Nestin	No
Xu et al., 2013 [41]	Allogeneic-human (healthy voluntary donors)	Embryo (allogeneic-human)	Dopaminergic neurons	Differentiation was first induced with a certain concentration of ascorbic acid, followed by normal human CSF treatment for inducing the differentiation of stem cells from the embryonic mesencephalon to dopaminergic neurons in order to obtain the maximum proportion of dopaminergic neurons	TH	No
Zappaterra et al., 2013 [42]	Allogeneic-rat	Embryo (allogeneic-rat)	Neuron-like cells	CSF from varying ages or conditions to investigate the biological activity of the CSF proteome on target cells	PH3, Tuj1, BRDU	Yes-animal experiments (rats)
Martín et al., 2006 [43]	Allogeneic-chicken	Embryo (allogeneic-chicken)	NSCs	FGF2 contained within chick E-CSF was involved in regulating the behavior of neuroepithelial stem cells	Genes	No
Parada et al., 2005 [44]	Allogeneic-chicken	Embryo (allogeneic-chicken)	NSCs	The expression of neuroepithelial genes in ESCs was affected by CSF	BRDU, genes	No
Kiiski et al., 2013 [45]	Allogeneic-human (healthy voluntary donors)	Embryo (allogeneic-human)	Neuron-like cells	Human CSF supported neural cell growth whereas artificial CSF was detrimental to the cells; human CSF promoted glial differentiation over neuronal differentiation	Nestin, MAP2, GFAP	No
Ma et al., 2013 [46]	Allogeneic-human (patients)	Embryo (allogeneic-rat)	NSCs	Adult CSF did not support neurogenesis from fetal rat NSCs	Nestin, NSE, GFAP	No
Buddensiek et al., 2009 [47]	Allogeneic-human (patients)	Embryo (allogeneic-rat)	Neuron-like cells	CSF inhibited the differentiation of ESCs into neurons but promoted their differentiation into glial cells	Nestin, GFAP, β-tubulin	No
Li, 2012 [48]	Allogeneic-rat	Endogenous neural stem cell (Allogeneic-Rat)	NSCs	After spinal cord injury, changes of the CSF components affected the proliferation and differentiation of endogenous NSCs	BRDU, Nestin	No
Liu, 2007 [49]	Allogeneic-human (traumatic brain injury patients), allogeneic-human (hydrocephalus patients)	Endogenous neural stem cell (Allogeneic-Rat)	NSCs	1) NSC could survive, proliferate and differentiate in both the traumatic bloody CSF and hydrocephalic clear CSF. 2) There was faster and higher percentage of adherent differentiation of NSC in traumatic bloody CSF than in hydrocephalic clear CSF. 3) The NSC differentiation types were different when induced by the traumatic bloody CSF and hydrocephalic clear CSF. NSC tended to differentiate into glial cells in traumatic bloody CSF, and into neurons in hydrocephalic clear CSF.	Nestin, NSE, GFAP	No
Teng et al., 2003 [50]	Allogeneic-rat (cerebral ischemia), allogeneic-rat (normal)	Endogenous neural stem cell (allogeneic-rat)	NSCs	CSF of cerebral ischemia rats promoted the survival of NSCs in vitro, and promoted the differentiation of NSCs into neurons and astrocytes	Nestin, NSE, GFAP	No
Nozaki et al., 1992 [51]	Allogeneic-human (subarachnoid hemorrhage patients)	Endogenous neural stem cell (allogeneic-human)	NSCs	The bloody CSF from subarachnoid hemorrhage patients was an effective stimulant for activating and promoting the proliferation and differentiation of endogenous NSCs	Genes	No
Haines et al., 2015 [52]	Allogeneic-human (multiple sclerosis patients)	Endogenous neural stem cell (allogeneic-rat)	NSCs	CSF from multiple sclerosis patients could affect the transcription in oligodendrocyte progenitor cells of NSCs	Genes	No
Peirouvi et al., 2015 [53]	Allogeneic-rat	Embryo (allogeneic-rat), endogenous stem cell (allogeneic-rat)	Neuron-like cells	The proportion of neurons and glial cells differentiated under different pathophysiological states from ESCs at different stages and hippocampal NSCs at different locations was different	Nestin, β-tubulin, GAFP, MAP-2, Genes	No
Delgado et al., 2014 [54]	Allogeneic-rat	Endogenous neural stem cell (allogeneic-rat)	Neuron-like cells	NO-preconditioned rat CSF promoted the proliferation and differentiation of the subependymal nerve cells	Nestin, BRDU, GAFP, genes	No
Cristofanilli et al., 2013 [55]	Allogeneic-human (multiple sclerosis patients)	Endogenous neural stem cell (allogeneic-human)	Neuron-like cells	CSF of patients with progressive multiple sclerosis promoted the differentiation of human NSCs into neurons and oligodendrocytes, but the ratio was different	MAPR, TUJ1, GFAP, genes	No
Buddensiek et al., 2010 [56]	Allogeneic-human	Endogenous neural stem cell (allogeneic-human)	Neuron-like cells	Under different conditions, CSF mainly promoted NSC gliosis, occasionally neuron-like differentiation was mainly observed	BRDU, GFAP, genes	No
Wang et al., 2016 [57]	Allogeneic-rats	Endogenous neural stem cell (allogeneic-rat)	Neuron-like cells	CSF containing traditional Chinese medicine induced the increment and differentiation of endogenous nerve cells, and the cell vitality and the disease resistance were stronger	Tubulin, GFAP, genes	No

*Aβ* amyloid beta-peptide, *BM* bone marrow, *BRDU* bromodeoxyuridine, *CSF* cerebrospinal fluid, *E-CSF* embryonic cerebrospinal fluid, *ESC* embryonic stem cell, *FGF2* basic fibroblast growth factor2, *Galc* galactocerebrosidase, *GBM* glioblastoma multiforme, *GFAP* glial fibrillary acidic protein, *GLP-1* glucagon-like peptide-1, *hAMSC* human amniotic mesenchymalstem cells, *hfNPC* human fetal neural progenitor cell, *IF* immunofluorescence, *IGF-1* insulin-like growth factor-1, *IHC* immunohistochemistry, *MAP2* microtubule-associated protein 2, *MAPR* membrane-associated progesterone receptor, *MSC* mesenchymal stromal cell, *N/A* not available, *NF* neurotrophic factor, *NSC* neural stem cell, *No* No transplantation, *NSE* neuron-specific enolase, *PH3* phosphorylated histone H3, *TH* Tyrosine hydroxylase, *Tuj1* neuronal class III β-Tubulin, *UCB* umbilical cord blood
